# Reinforcing nature-based solutions through tools providing social-ecological-technological integration

**DOI:** 10.1007/s13280-022-01801-4

**Published:** 2022-10-26

**Authors:** Thilo Wellmann, Erik Andersson, Sonja Knapp, Angela Lausch, Julia Palliwoda, Jörg Priess, Sebastian Scheuer, Dagmar Haase

**Affiliations:** 1 Landscape Ecology Lab, Geography Department, Humboldt-Universität zu Berlin, Unter den Linden 6, 10099 Berlin, Germany; 2 Department of Computational Landscape Ecology, UFZ – Helmholtz Centre for Environmental Research, Permoserstr. 15, 04318 Leipzig, Germany; 3 Ecosystems and Environment Research Programme, University of Helsinki, PB 65 (Viikinkaari 1), 00014 Helsinki, Finland; 4 Stockholm Resilienc Centre, Stockholm University, Albanovägen 28, 106 91 Stockholm, Sweden; 5 Unit for Environmental Sciences, North-West University, Private Bag X6001, Potchefstroom, 2520 South Africa; 6 Department of Community Ecology, UFZ – Helmholtz Centre for Environmental Research, Theodor-Lieser-Str. 4, 06120 Halle (Saale), Germany; 7 German Centre for Integrative Biodiversity Research (iDiv) Halle-Jena-Leipzig, Puschstraße 4, 04103 Leipzig, Germany; 8 Department of Ecology, Ecosystem Science/Plant Ecology, Technische Universität Berlin, 12165 Berlin, Germany; 9 Humboldt-Universität zu Berlin, Unter den Linden 6, 10099 Berlin, Germany

**Keywords:** Climate change adaptation, Functional diversity, Nature-based solutions (NbS), Remote sensing, Resilience, Social-ecological-technological systems (SETS)

## Abstract

While held to be a means for climate change adaptation and mitigation, nature-based solutions (NbS) themselves are vulnerable to climate change. To find ways of compensating for this vulnerability we combine a focused literature review on how information technology has been used to strengthen positive social–ecological–technological feedback, with the development of a prototype decision-support tool. Guided by the literature review, the tool integrates recent advances in using globally available remote sensing data to elicit information on functional diversity and ecosystem service provisioning with information on human service demand and population vulnerability. When combined, these variables can inform climate change adaptation strategies grounded in local social–ecological realities. This type of integrated monitoring and packaging information to be actionable have potential to support NbS management and local knowledge building for context-tailored solutions to societal challenges in urban environments.

**Supplementary Information:**

The online version contains supplementary material available at 10.1007/s13280-022-01801-4.

## Introduction

Urban vegetation and the ecological functions it provides are important for the wellbeing of people in cities (Andersson et al. 2014, 2019). However, the urban environment poses unique challenges and constraints to the growth conditions of plants, now exacerbated by climate change. Increasingly ambitious programs and strategies for greening cities make ecosystem viability and resilience central concerns, which have only partially been addressed (Andersson et al. 2017, 2019). In the current work with nature-based solutions (NbS)—defined as actions for enhancing or creating vegetation structures to ensure ecosystem service provisioning and human wellbeing (Cohen-Shacham et al. 2016)—the resilience and stability of the biological foundation of NbS are neglected compared to their ‘optimal provisioning’ of ecosystem services in a given situation.

Resilience is essential for understanding NbS functional performance over time, i.e., their ability to continue to deliver vital functions over longer periods despite disturbances and changing climate (Weise et al. 2020). Diverse arrays of NbS types and management arrangements are hypothesized to provide response diversity and thus resilience, at the level of individual NbS as well as the aggregate city level. Still, many articles about ecosystem-based adaptation do not offer clear, actionable management suggestions for resilient NbS in cities (Brink et al. 2016; Wickenberg et al. 2021).

NbS in cities have social (users and managers), ecological (the green and blue structures providing or embedding specific NbS) and increasingly often technological dimensions (e.g., built components or sensors for monitoring) (Li and Nassauer 2021). The social-ecological-technological systems (SETS) they form challenge current governance and engineering approaches (Dhakal and Chevalier 2016). NbS such as green roofs or constructed wetlands tend to be dispersed throughout a city, across space, land ownership, and jurisdictions with differing urban challenges being addressed and ecosystem services provided (Almenar et al. 2021). In such cases, collaboration and information exchange between different stakeholders is key for NbS viability and upscaling (Tzoulas et al. 2021; Venter et al. 2021; Wickenberg et al. 2021). Thus, overcoming limitations imposed by sectoral, comparatively narrow, and often actor-specific processes is essential to support NbS implementation, as well as for building resilience around NbS management (Croeser et al. 2021; Lin et al. 2021). To overcome this, we hypothesize that social-ecologically framed and informed technology can serve as an instrumental link, enabling alternative, accessible and feasible pathways that support a more equitable urban system.

The goal of this study is to prototype a decision-support tool for climate change adaptation. This is done by integrating knowledge of the local social-ecological system, expertise in information technology (IT), and environmental monitoring data. We begin the article by describing the case of tree-based NbS under pressure from a climate change-induced drought to highlight some of the challenges that resilience-building around NbS needs to overcome. We then analyse, based on a focused review of the scientific literature on solutions using IT to connect social-ecological realities, the factors that are held to make IT-wired NbS resilient and just. The findings from the review are used to guide the design of a remote sensing-based decision-support tool for producing actionable knowledge and identifying alternative climate change adaptation pathways. We then use the case, the review, and the prototype tool to reflect on next steps and potential applications.

## The case: managing tree-based NbS during climate change

Many cities in Europe rely on tree plantings to face climate change―predominantly heat waves (Kabisch et al. 2016). By providing cooling, air purification, air moisture, and carbon sequestration (Konijnendijk et al. 2006), trees are an effective strategy to mitigate and adapt to climate change. Trees are considered the largest provider of ecosystem services among NbS in cities (Nesshöver et al. 2017; Tzoulas et al. 2021).

However, trees as parts of ecosystems and living nature also depend on water availability, air temperatures within their range of tolerance, and insolation (Nock et al. 2013). Particularly in cities, trees face compacted, partly contaminated soils and limited effective rooting depths, and are thus exposed to stresses such as high susceptibility to pests, leading to limited vitality (Sjöman et al. 2018). The recent heat waves in central Europe have shown that NbS in cities cannot solely rely on natural processes (Haase and Hellwig 2022). Therefore, support is needed if ecosystem service delivery is to be maintained as envisaged in the NbS.

**Fig. 1:**
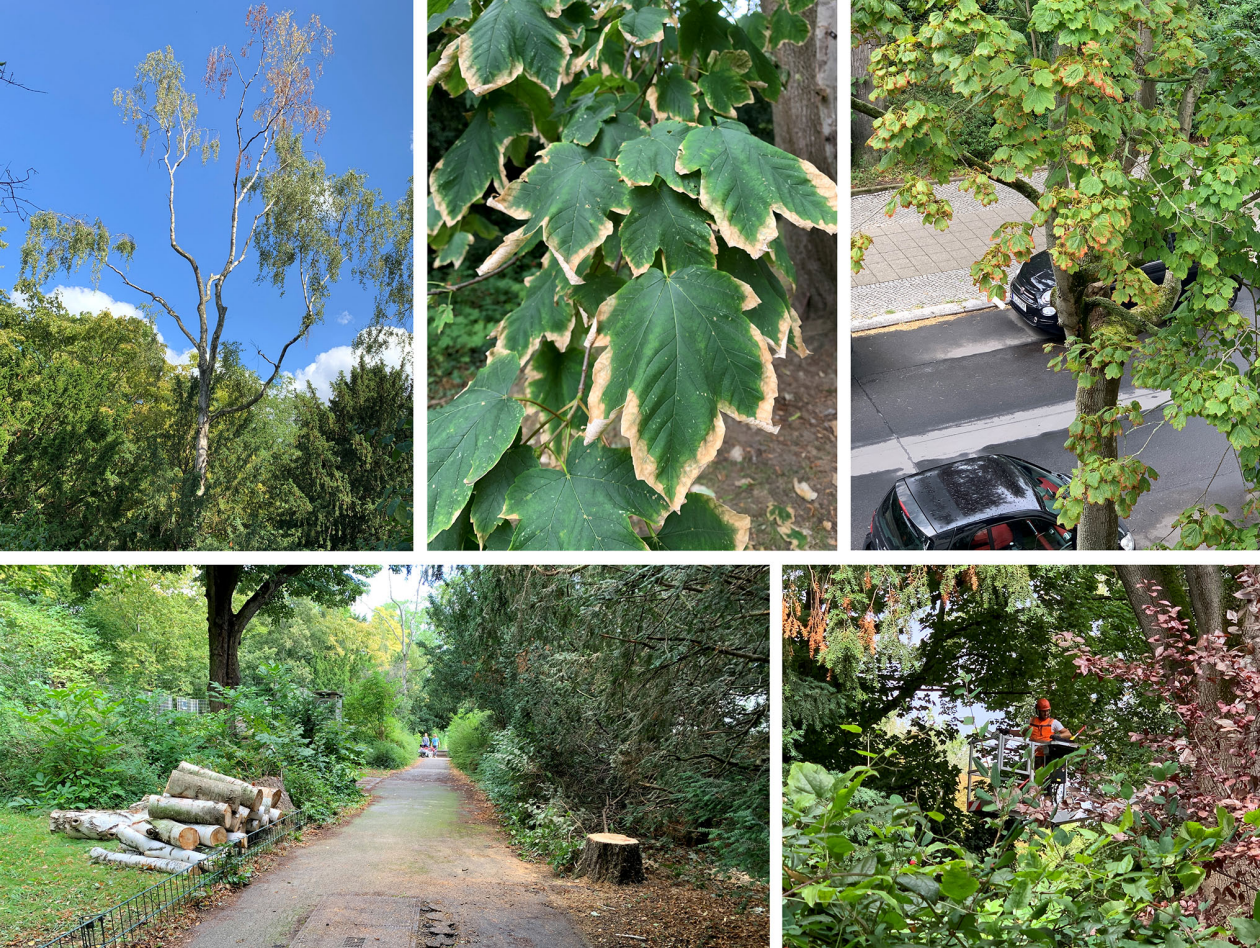
Heat and drought induced changes in canopies of urban trees in the upper row. Consequences of those damages in form necessary tree care and felling in the lower row. Photos: Thilo Wellmann

Heat or drought-caused tree damages such as senescence, leaf abscission or branches and whole trees dying off (Fig. 1) reduce benefits like shading, cooling or carbon sequestration. As trees are long-lived organisms, long-term intentional planting and management strategies are needed. There are two main strategies for addressing these circumstances. The first is to make tree communities less vulnerable by increasing the diversity of those communities, with a focus on presumably more robust and stress-tolerant species. The second is to adapt and intensify the management of existing tree assemblages. For the former, there is ample evidence that vegetation structures rich in species, functional effect and response traits, phylogenetic relationships, or genetic diversity are less susceptible to change, and therefore more resilient in times of change and uncertainty (de Bello et al. 2021). This helps ensure that some benefits of NbS will be delivered even if single species fall ill to a pest or suffer from inadequate growth conditions. Concerning the latter strategy, cities tend to have limited natural or financial resources, which means that approaches need to be targeted and effective. The two strategies are mutually inclusive, and both can be strengthened by the involvement of local stakeholders.

To exemplify: Located at the transition between continental and maritime climate, Leipzig (51° 20' N, 12° 23' E), a German city of around 600,000 inhabitants, receives around 530 mm rainfall per year. There is substantial seasonal and annual variation in precipitation (Deutscher Wetterdienst 2022). Leipzig’s parks and brownfields are valued for providing regulating services (e.g., shade provisioning) and offering space for ecological diversity in close proximity to people’s homes (Palliwoda and Priess 2021). Tree-based greening has been a widespread and seemingly successful NbS during both population shrinkage and recent re-growth (Rink and Schmidt 2021). However, the current scarcity of rainfall in some years has increased the mortality of trees.

Heat stress can be intense and is associated with high vulnerability, particularly since the population in Leipzig is aging, with some districts featuring up to 90% senior citizens (Scheuer et al. 2021). To describe the vulnerability and risk patterns of the local community (Yang et al. 2019), we acquired two datasets from the cities open data portal (https://opendata.leipzig.de), namely population density and share of senior citizens (age above 65 years) per district (Stadt Leipzig). Vulnerabilities and risks associated with extreme weather events are highest in locations with disproportionally high shares of elderly citizens (Nayak et al. 2018). At these locations, failure to deliver ecosystem services such as cooling or shade provisioning by trees would have particularly severe implications.

**Fig. 2:**
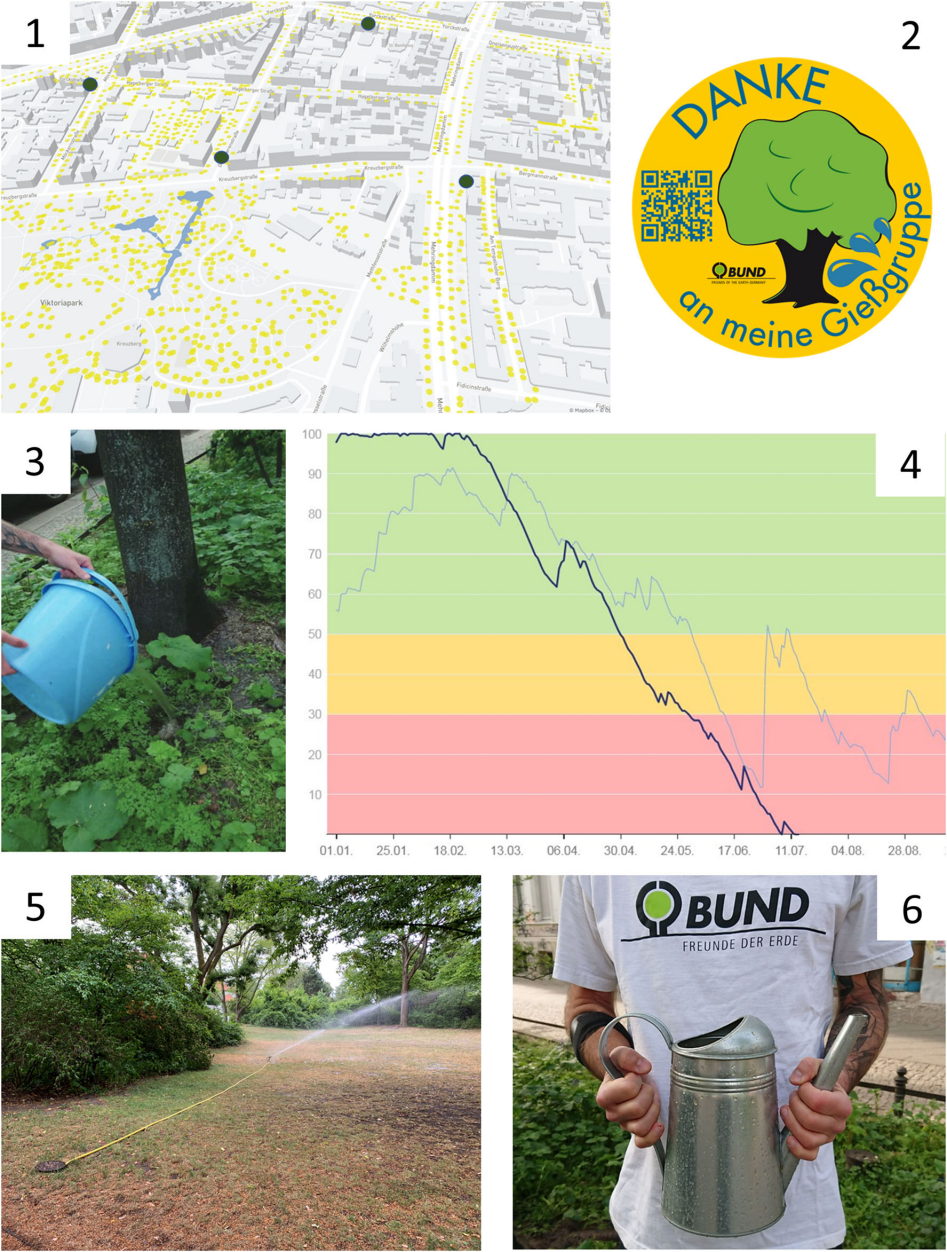
Collage of social- ecological and technological means for knowledge dissemination around reducing drought stress in tree-based NbS (nature-based solutions). (1) Map showing location of street trees with current distribution of precipitation (in yellow) together with locations of public water source (in blue). (2) Symbol of an initiative led by a local NGO (BUND-Berlin) for bringing residents together for watering local trees in joint actions and (3) a handbook advising them how to collect grey-water for doing so. (4) Chart showing soil humidity with colour indications representing urgency for watering trees, carried out in a large scale from tap water in photo 5. (6) An NGO advocating for people to become stewards for tree watering. Image Sources: (1) CityLab Berlin: www.gießdenkiez.org; (2,3,6) BUND-Berlin; (3) https://www.bund-berlin.de/fileadmin/berlin/publikationen/Naturschutz/baeume/Begruenen_von_Baumscheiben.pdf & (2,6) https://www.bund-berlin.de/mitmachen/aktion-baeume-giessen/; (4) Stadt Berlin: Bodenfeuchteampel; (5) Thilo Wellmann) All online material accessed July 2022

Activating and interconnecting local support networks across stakeholder groups have the potential to reinforce NbS. This requires access to relevant information and platforms for civic engagement e.g., by local NGOs and central management departments in cities (Andersson et al. 2014). Examples of social- ecological- and technological (SET) approaches towards activation and exchange around trees under drought stress are collected in Fig. 2. Working in this direction, the city runs a street tree cadastre and provides a web-based tree location map to inform the public on new plantings and tree stewardship projects (Programme ‘Baumstarke Stadt’; https://www.leipzig.de/freizeit-kultur-und-tourismus/parks-waelder-und-friedhoefe/spenden-und-patenschaften/baumstarke-stadt). The city is also developing a new ‘Masterplan Green 2030’ where trees play a core role (https://www.leipzig.de/freizeit-kultur-und-tourismus/parks-waelder-und-friedhoefe/masterplan-gruen).

## Linking SETS dimensions to reinforce NbS

### Focused review: Options to reinforce NbS

NbS are vulnerable towards climate change-induced extreme events in many cities. As urban plants are faced with many pressures and constraints like reduced water availability due to sealing by impervious materials, it is challenging to integrate resilience into urban NbS as is done for non-urban NbS. Reinforcement of classic grey solutions tend to rely on technical expertise and local to national authorities and have therefore a long tradition in technological expertise around them (Dhakal and Chevalier 2016). Working within the intertwined and distributed nature of urban NbS, the knowledge of appropriate technological means of supporting their functioning is much less. We draw from the SETS framework that expanded connections and exchange between diverse stakeholders is a potential route of creating stronger connections in and around NbS (McPhearson et al. 2022).

We therefore ask; are there exemplary interfaces for us to learn from that involve social- ecological- and technological aspects to enable a broader stakeholder contribution and involvement around NbS? We paid special attention to IT-based approaches and the focused review assessed current evidence and best practices for enabling integrated, IT-mediated options for NbS management. We assessed what made the solutions successful and to what degree NbS were reinforced and potentially resilience was built around them. These tentative insights were then used as a base reference for our decision-support tool for supporting interfaces around the management of NbS in our case city Leipzig. The query was performed on 01 November 2021 and resulted in 29 articles, of which nine did not meet our scope or were unavailable. The remaining 20 articles were analysed using a review protocol (detailed review methods and bibliographical information can be found in the supplementary material).

### SET-interfaces for reinforcing NbS

**Fig. 3:**
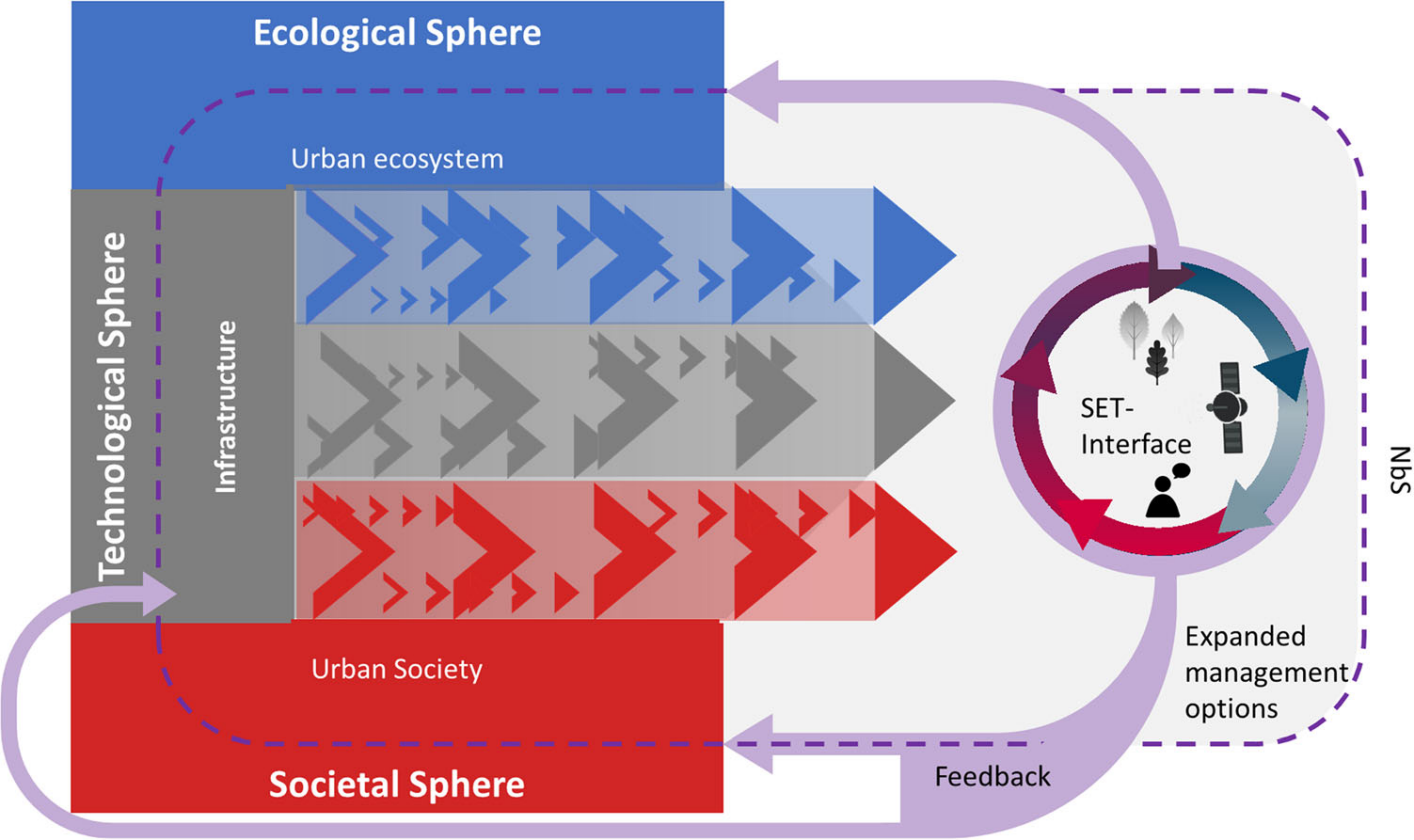
Concept of a Nature-based Solution (NbS) that is reinforced though a connected social-ecological-technological-support system. Points of connection, i.e., interfaces, offer potential for advancing the scope of action around NbS management by providing knowledge exchange, facilitating joint actions, and giving meaningful feedback to specialists in and outside the NbS (e.g., feedback to a nursery, based on tree species functioning and stakeholder preferences) (some icons desgned by Freepik.com)

The analysed literature provided several indications how NbS can be reinforced through connecting them better with their social-ecological system (Fig. 3). Evidence for this was drawn from 20 studies reporting benefits, risks and additional pathways realised through establishing connections, i.e., interfaces between two or more SETS dimensions. The articles were published between 2014 and 2021 across 17 journals and study different types of system couplings (See Table S1 in the supplementary material for bibliographical details). Socio-technological interfaces for NbS management can be online platforms for learning, sharing and decision-support (see e.g., Afzalan and Muller 2014). Eco-technological interfaces are exemplified by solar panels for habitat diversification (Nash et al. 2016) or environmental data collected by sensor networks (Bellamy et al. 2017). Social-ecological interfaces can be different mechanisms for local knowledge integration like citizen science or human-biodiversity interactions (Fastenrath et al. 2020).

Through the establishment of such connections, additional opportunities opened up for management in the small subset we reviewed. The majority of studies report that in distributed urban NbS, information flow and knowledge sharing are of importance (Møller et al. 2019). Exemplary types of IT for stakeholder support include web-based apps, smartphone apps, GIS-platforms, larger cloud computing systems for data analysis, or video conferencing suites. These suites could link between and within stakeholder groups allowing for both bottom-up and top-down pathways in the reviewed examples (Møller and Olafsson 2018; Taylor et al. 2021).

More precise spatial targeting of management efforts based on the input from local communities was one of the more frequent features of the 20 studies. Dhakal and Chevalier (2016) reported that interactive tools in their case allowed the distribution of service-providing units to better mitigate climate change impacts, while Venter et al. (2021) achieved better priority area designation. In the study by Taylor et al. (2021), residents were included in questions of park location and design through their tool.

A second group of papers reported how individuals, departments, and citizens were connected through various IT developments. Møller et al. (2019) show that engagement of the private sector, which was previously not integrated into green management, was facilitated by a tool. Afzalan and Muller (2014) show that IT could spur in-person meetings between previously unknown persons contributing to co-management and co-creation of NbS. Between the reviewed papers we found diverse reporting on the range of stakeholders additionally incorporated by the knowledge interfaces, from very specific targeting of citizens to successful integration of formerly overlooked communities. In the case study by O’Donnell et al. (2018), IT helped as a visual communication tool promoting collaborative learning in the adaptation process. Hasala et al. (2020) report that improved exchange around NbS empowered marginalized groups in a decision process. Further, we found examples where switching between management strategies were made easier through better connected SETS dimensions (O’Donnell et al. 2018; Leonard et al. 2019; Møller et al. 2019).

Continued impact assessments are valuable for continued engagement. According to Hasala et al. (2020), especially less deterministic and more open governance processes require an evidence basis that is up-to-date and trustworthy. Those can be visioning and scenario development, which are an important foundation for co-creation and co-management.

While we found evidence that IT can help in connecting stakeholders, our focused review targeting such tools cannot support any claim that IT is necessarily the best and most widely used means for connecting urban SETS. Generally, such tools should not be seen as replacing more traditional approaches to participation process, but rather as adding to them (Gulsrud et al. 2018). Heavy reliance on digital tools may also lead to the exclusion of people not having access to them and can also lead to decoupling (Finewood et al. 2019). Therefore, tools that work on- and off-line as well complementary in-person meetings in line with democratic procedures are key to broaden engagement around NbS.

Table 1 lists the factors elicited from the reviewed literature corpus for successful knowledge interfaces, which were organized under three themes: common ground, diverse knowledge and continuous performance evaluation. We connect those prerequisites to facets of the tools that, according to the authors, enabled the reported benefits. These findings serve us as an interim learning outcome from the review and are, together with the social-ecological case description, the basis of the tool developments in the following chapter.

**Table 1:** Lessons learned from the focused literature review on how interfaces can connect the social-ecological-technological (SET) dimensions in nature-based solutions (NbS) management for community involvement. We show how the lessons learned can be translated to good design practices for information technology (IT) based tools

Theme	Lessons learned for SET-interfaces	Lessons learned for IT design
*Common ground*	*Place-based approaches* are valuable as all NbS—and most crucially their benefits—manifest themselves at some physical place (Møller et al. 2019). Linking structures and ecosystems services to places and people is a crucial aspect of public engagement regarding NbS management (Taylor et al. 2021)	Digital tools need to *contextualise* environmental data regarding performance, ecosystem service provisioning, and resilience in spatially explicit ways (Fu et al. 2019; Leonard et al. 2019)
	*Local knowledge integration,* especially that of marginalised communities, can lead to locally adapted NbS structures that are desired (Bellamy et al. 2017; Hasala et al. 2020)	
*Diverse knowledge*	*Inclusive language* can facilitate better integration of diverse backgrounds and thus allow for amplified exchange between stakeholders regarding NbS (Fastenrath et al. 2020). Interfaces need to speak to people with different backgrounds and to non-experts	*Visualisation capabilities* are important for IT seeking to connect NbS. *Lowering entry* barriers is important meaning that visualisations can be (partially) used offline and in non-digital form to ensure inclusive visioning (Gulsrud et al. 2018; Møller and Olafsson 2018)
	A key step towards just NbS is *effective and inclusive visioning* (Newton and Frantzeskaki 2021). This can enable stakeholders to select appropriate pathways and more integrated measures (O’Donnell et al. 2018)	
*Feedback*	*Acknowledging the contributions of the community* is key for further spurring the desire to contribute to NbS (Fastenrath et al. 2020). Seeing the impacts of one’s action is important for continuous engagement in a multi-stakeholder system (Møller et al. 2019)	*Data* need to be of high quality stemming from multiple sources and be trustworthy (Møller et al. 2019), making the content more important than the tools design (Ugolini et al. 2015). A data type of special interest is monitoring data like remote sensing data as it allows for spatio-temporally continuous representations of multiple facets of urban SETS (Bellamy et al. 2017; Leonard et al. 2019)
	*Transparent actions and continuous information flow* with direct links between stakeholders and feedback allows for furthering the engagement and creates a situation where differing opinions and realities can be heard (Møller et al. 2019)	

## Operationalisation: NbS incorporating remote sensing, functional diversity and population vulnerability

**Fig. 4:**
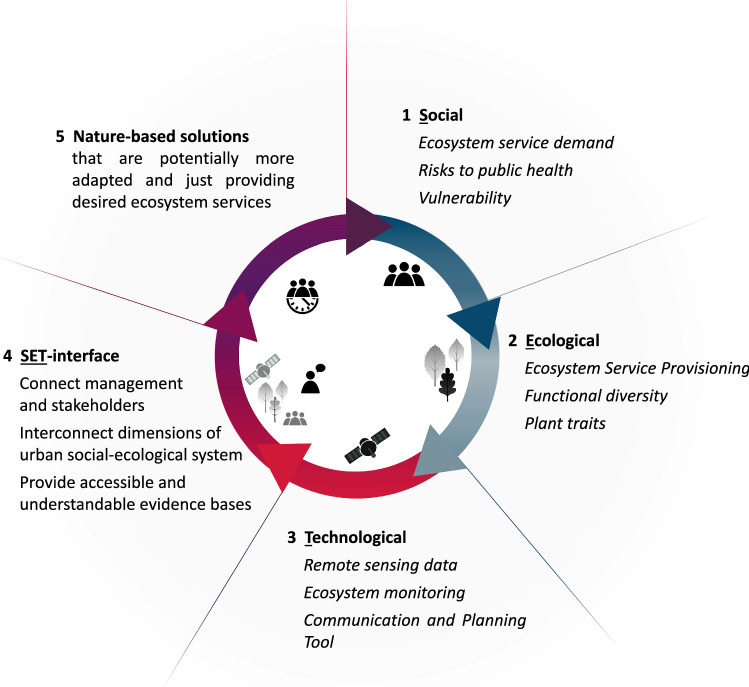
An exemplarily social-ecological-technological (SET) interface connecting aspects important for climate change adaptation. *Text in italic represents the means we are undertaking in the study* (some icons designed by Freepik.com)

“The case: tree-based NbS management during climate change” section showed that Leipzig exhibits growing risks as population density and vulnerability is growing. At the same time, climate change-induced heat waves are on the rise, making more frequent assessment cycles and faster information flows vital for decision-making. From the focused review, we take that tools with low entry barriers showing regularly updated and trustworthy data can be interfaces in such a social-ecological setting. In this section, we thus develop a prototype interface based on plant traits to describe aspects of functional diversity in tree communities in combination with remote sensing data for continuous monitoring (Fig. 4).

### Methods and indicator description

#### Plant species mapping and trait data

Using trait-based concepts in urban forestry can be beneficial, as such frameworks provide linkages to ecological functioning and ecosystem services as well as aesthetic considerations (Andersson et al. 2021). “A trait is any morphological, physiological or phenological feature measurable at the individual level, from the cell to the whole-organism level.” (Violle et al. 2007, p. 884). The number and range of functional traits in turn determines the functional diversity of an ecosystem (Díaz and Cabido 2001). As there is no single trait that sufficiently relates to tree species selection in the urban environment (Watkins et al. 2021), our proposed indicator accounts for variations in multiple such traits relevant for plant functioning.

We mapped present tree species in randomly located 15 × 15 m plots featuring varying degrees of management intensity, ranging from brownfields with complete absence of management to heavily used, planted, and well-maintained urban greenery in the form of parks. In so doing, all woody plants above 2 m were recorded. The campaign took place in 2017 in 18 brownfields and 18 parks (Palliwoda et al. 2020).

Based on the species mapping, we acquired trait data from the TRY trait-database, representing the largest repository of functional plant characteristics (Kattge et al. 2020). If multiple entries for a single species were present, we calculated the median value for the respective trait. Thereof, we calculated the range in all numerical traits as the indicator for plant functional diversity for each of the vegetation communities (Díaz and Cabido 2001).

Traits were select following three steps: First, based on Homolová et al. (2013), recommending that plant traits related to leaf biochemistry, photosynthetic processes, and canopy structure are best detectable by optical remote sensing. Second, looking at the species present in our study and the data available for them in the TRY database, we sorted for those traits that had the most information for the tree species present in our research plot. Third, between the remaining traits we searched in a non-formalised way for literature showing satisfying co-relations between the traits’ components and optical remote sensing. Based on this, we selected leaf area, photosynthesis rate per leaf area, leaf nitrogen content per leaf dry mass, and leaf dry-matter content as the most relevant and promising traits for our analysis.

#### Remote sensing data and processing

One well established data source for cost-effective and repeated analysis of vegetation functioning covering large spatial areas is satellite-based remote sensing (Lausch et al. 2018). Remote sensing data provide a window in the functional diversity of tree-based ecosystems (Schneider et al. 2017; Zheng et al. 2020; Zhang et al. 2021), and are promising for the monitoring of urban green and its benefits (Andersson et al. 2020; Chrysoulakis et al. 2021; Shahtahmassebi et al. 2021).

We acquired PlanetScope data featuring a spatial resolution of 3 m and four spectral channels, namely red, green, blue, and near-infrared (Planet Labs 2022). A total of 13 scenes were analysed, taken during the growing season between March to October (and one additional winter scene), stemming from 2017 (average precipitation), complemented with acquisitions from 2018 (dry summer). Based on surface reflectance values, the Normalized Difference Vegetation Index (NDVI) was calculated alongside a principal component analysis (PCA) for each of the individual images. The NDVI is a band relational index allowing the detection of tree vitality and changes therein, e.g., as response of trees to drought (Lloret et al. 2007).

The NDVI and the PCA were used to calculate the trait variation indicators. For doing so, we calculated texture metrics based on the grey level co-occurrence matrix (GLCM), expressing the spatial variation in each of the NbS (Xie et al. 2023), as suggested in Haralick et al., (1973). The remotely sensed spatial variations in each of the NbS was then integrated over time to derive the annual amplitude in variations for every site. This was done by subtracting the minimum in spatial variation from the maximum. This is needed to eliminate the spatial variability caused by built features that remain stable over the course of a growing season and to account for the fact that, depending on the plants, differences between them are amplified in certain stages of the phenological cycle (Wellmann et al. 2018). As a last step, bringing trait data and remotely sensed information together, we correlated the computed amplitudes in annual spectral variation with the range in the four selected numerical traits.

### Societal and ecological indicators for NbS management

#### Functional plant diversity indicator

Functional diversity among the plots varied greatly. We found NbS with differing degrees of functional diversity resulting from varying planting strategies together with natural processes in our case study city. Many plots showed a lack in diversity in all four traits, thereby highlighting NbS systems of amplified vulnerability. We found statistically significant relationships between the remote sensing indicators and diversity in four key traits in the analysed vegetation communities (Table 2), with the annual range in those texture metrics being positively correlated to the range in traits in the analysed plots.

**Table 2:** Correlation between three remote sensing-derived texture metrics describing the range in spectral diversity with diversity in four traits. On the remote sensing side, the NDVI (Normalized difference vegetation index) and a PCA (Principal component analysis) were used as input bands. The correlation coefficient is Pearson's *r*

Remote sensing	Texture metric	Trait	Corr. coefficient
NDVI	Entropy	Photosynthesis rate per leaf area	0.55
		Leaf area	0.55
PCA band 1	Contrast	Leaf nitrogen content per leaf dry mass	0.34
	Variance	Leaf dry-matter content	0.32

#### Performance response monitoring

The response in performance to natural forcing—a drought in this example—can also be analysed with the proposed methods and datasets. For this, we contrasted the NDVI of two late-summer images from two consecutive years, of which the second was a hot and dry year. The NbS systems showed a reduction in NDVI ranging from 9 to 50% suggesting losses in photosynthesis capacity in all sites. This shows that in times when ecosystem services such as cooling and shading are of outmost importance, those benefits may not be provided by urban NbS in its current configuration and the current support system, potentially causing lasting damages to trees.

#### Population density and risk

Population density and risk associated with higher proportions of senior citizens varied substantially between the districts of Leipzig. This means that both ecosystem service demand, as well as the risk associated with failed ecosystem service delivery differs substantially between the different NbS systems. In the majority of districts, there is an ambivalent picture, while the demand for NbS is highest in the centrally located areas, the risk is highest in the surrounding districts with medium population density. Exceptions are prefabricated housing districts featuring both high demand and risk for services. The proportion in senior citizens can reach up to 89% here (Grünau), whereas the district with the lowest risk only features a share of 9% in senior citizens (Lindenau). Thus, demand and risk were unevenly distributed in this medium-sized city.

### Mapping and prioritising the scope of action around NbS management

**Fig. 5:**
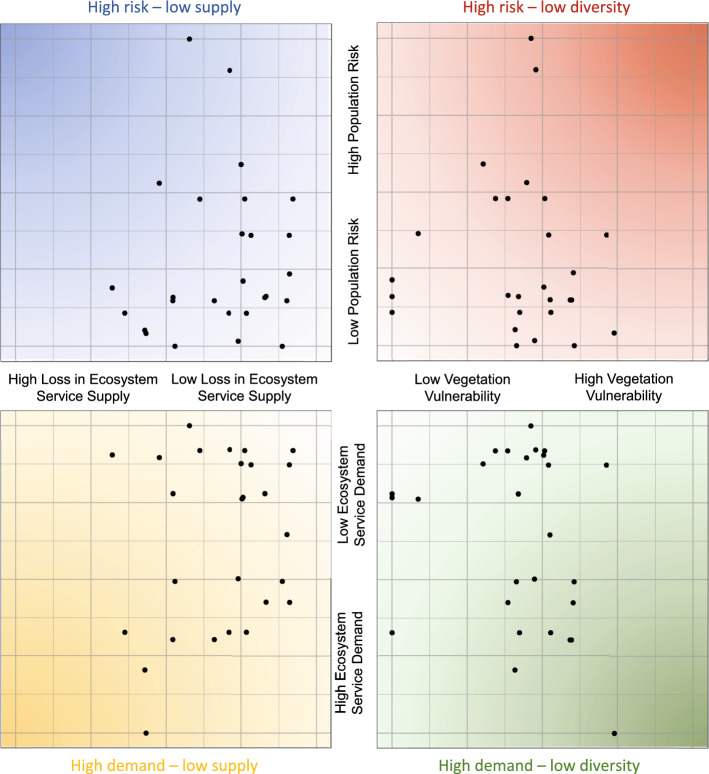
Decision-support system for balancing climate change adaptation goals. Each of the black dots represents one nature-based solution (NbS), for which losses in ecosystem service and the vulnerability in their tree communities are shown. The ecological variables are set in to context with the social surrounding of the NbS, namely population density and public health risks if services are not provided. The closer to the inside the NbS is located in the feature space, the better the system performs in the respective indicator combination. See Fig. 6 for an exemplifying adaptation goal

This chapter develops a case-based support tool focussing on knowledge integration and dissemination around climate change adaptation pathways (Fig. 5). It integrates remote sensing-derived indicators with data on social vulnerability and population density, as context for challenge-oriented NbS evaluation. A NbS can function in one place, as it is a solution to the given problem setting, but the same NbS would not be seen as functional given a different problem setting. We assessed the proportion of the population that is vulnerable to heat and population density as variables to which NbS functioning can be related. These two variables allow different adaptation goals to be contrasted and balanced—e.g., serving the greatest number of people or serving the most vulnerable people, with ecosystem services to reduce the risk of fatalities. As both goals are obviously valid and the means to reach them can differ, involvement and democratic procedures are required to balance management type, intensity, and resources. In this direction, the tool seeks to provide an evidence basis and a starting point of discussion for other stages of management development, such as visioning and scenario development.

On the ecological side, we assessed functional diversity in trees as a representation of NbS vulnerability. The second plant indicator is the performance response of the NbS to a drought, representing changes in ecosystem service provisioning (only negative changes). All indicators were classified around their median and aggregated to NbS level, meaning each point in Fig. 5 represents one park or vegetated brownfield. We exemplify how adaptation measures can be influenced by their social surrounding with the following three examples:

#### NbS in areas of high population risk

In areas of amplified risks, like Grünau, service provisioning needs to be kept high—especially during heat waves—for protecting vulnerable population groups though cooling. Means to achieve this could be providing additional water supply by either retention, below-surface storage or sorptive substrates, as watering with tap water seems undesirable in a long-term climate change adaptation scenario. Such watering could amplify functioning in heatwaves and therefore lesser attention must be put on functioning by redundancy and diversity. Relating the risks in the local population to losses seen in ecosystem services can thus be a way for managers to assess where to act during a drought and heatwave first and where additional grey support infrastructure is needed.

#### NbS in high density dwellings

**Fig. 6:**
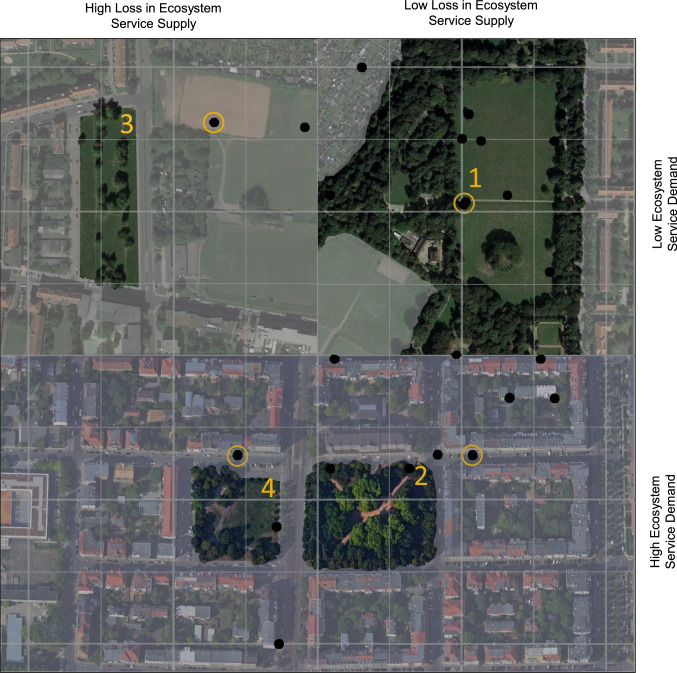
Adding remote sensing images directly to visualisations can help to set NbS into (spatial)context, here with the example of the built density around them. Based on such representations, management choices and prioritisation can be impacted, for instance regarding supply or demand-based prioritisation. The regarded NbS are highlighted with the location of the NbS in the feature space. The four NbS are: (1) Mariannenpark, (2) Heinrich-Schütz-Platz, (3) Brownfield Saalfelder Straße and (4) Alexis-Schumann-Platz

NbS in areas with high population density like the Südvorstadt (Fig. 6), which in turn feature comparably low shares in vulnerable population groups, are equally important. Recreational functions can be seen as a central solution to dense housing offering no private green. Here an intermediate strategy of combining diversity with other support systems for water provisioning could be desirable. Slight reduction in cooling rates could be accepted as recreational functions might still be given with reduced water availability.

#### NbS in areas of low population density

Low-density areas like single-family homes feature more private green and the least amount of usage pressure. Here, co-benefits to nature can be seen as the primary solution to increase urban biodiversity, and functional diversity-driven approaches can be desirable. In such a case biodiverse NbS can be seen as functional even though their ecosystem service provisioning might be reduced while non-diverse NbS can be seen as (partially) unfunctional even though services are provided.

#### Balancing NbS adaptation pathways

As many NbS are multifunctional, local decision capabilities and preferences from residents need to be factored in greatly if primary functions of NbS are elicited. Synergies between social and ecological variables should be elicited, for instance NbS in and around the local district of Grünau serve a comparatively high number of both total and vulnerable residents.

The problem-oriented nature of NbS, providing specific solutions makes clear that neither performance, nor diversity ‘maximisation’ are necessary leading to positive outcomes. More drought-adapted species could reduce cooling in heat waves as they are adapted to lower water consumption. Built solutions supporting lesser adapted species could be more beneficial here. Targeting such investments in key areas is thus important.

No adaptation pathway is objectively superior, making negotiations and balancing regarding the given social-ecological-technological setting a necessity for decision-making in planning. Further, some aspects of diversity and performance increases might not be possible due to various social and health-related constraints in cities. For instance, there might be areas where a specific design is desired featuring specific tree-traits reducing the available range of functional diversity. Examples are street trees where law (in Germany) requires only one species to be planted along a road for visual continuity, or certain species are preferred as they shape the identity of a place (e.g., *Tilia* in Leipzig). Further, functional diversity might lead to impeded visibility or a highly allergenic species. Here, different adaptation measures like water retention or bioswale systems might be needed supporting specific designs.

Specifically, for the city of Leipzig, the previously explored options would need to be integrated into its green planning, namely the Master Plan Green 2030 and a map featuring at a minimum the two risk categories mentioned would need to be created. What is more, the digital version of the Master Plan will need an updating protocol and procedure to regularly monitor the risk state. This will include the processing of remote sensing data implemented in the municipal budget. Further, low entry barriers to access the risk maps via an app or a freely accessible portal are desirable. Developing and reshaping the visual appearance of the tool in this direction will be brought further in workshops around NbS functioning.

## Synthesis: SET-interfaces for NbS under climate change

Coupling, and if needed, rewiring the SETS dimensions becomes especially important in times of accelerated climate change characterised by increased uncertainty and vulnerability (Meerow et al. 2016). When investigating how cities coped with a drought, Buurman et al. (2017) found that an insufficient number of countermeasures were available for responding to this type of challenge. We focussed on trees in NbS, as they provide the vast majority of regulating services (Almenar et al. 2021) and health benefits (Marselle et al. 2020). We therefore searched the literature for interfaces coupling social- ecological- and technological facets for improved tree management in NbS.

We found evidence that carefully crafted and adapted IT can support interfaces within a social-ecological system. This is highlighted by our case of a hot drought, showing that without interconnected support systems, some ecosystem services will not be delivered in times when they are needed most and where they are needed most. Working towards greener and more resilient urban futures, NbS take a central role, seeking to provide multiple benefits for climate change adaptation (Somarakis et al. 2019) by explicitly considering the societal setting the ‘solution’ is embedded in (Almenar et al. 2021). Considering desired expansions of NbS in cities for delivering further benefits to residents and biodiversity amplified communication and knowledge exchange is needed.

Working in this direction, incorporating feedback and performance evaluation is key for continuous actions and learning in a multi-stakeholder governance system (Dumitru et al. 2020). It enables planners, policy analysts, land-owners and other stakeholders to actively follow the changes brought about by their investments or actions and can further discussions regarding climate change or tree management (Afzalan and Muller 2018). Monitoring and assessing success and failure of the NbS—trees in our case—enables learning and thus options for upscaling (Werners et al. 2021). In the reviewed literature monitoring data were lacking but highlighted as desirable. Making people more engaged is key in furthering future successful climate change adaptation (Egerer et al. 2021).

### Information technology can be a connector and a barrier in urban climate change adaptation

There are examples where introducing IT to urban policy and planning can connect stakeholders around NbS, as the focused review shows. Connecting, translating and making different knowledge actionable is key to successfully work towards resilient polycentric forms of NbS management (Tengö et al. 2017; Zingraff-Hamed et al. 2021; Kabisch et al. 2022). Following Partelow and Winkler (2016), we regard three knowledge types occurring in social-ecological systems, namely system knowledge, target knowledge and transformative knowledge. It is common that not all stakeholders possess all knowledge types. Systems knowledge represents a significant barrier in management seeking the integration of technology. Transformative knowledge in turn can exist in local activist groups as innovative and transformative practices are not common amongst planning professionals (see an example from Germany in Othengrafen and Levin-Keitel (2019)).

Therefore, approaches connecting those knowledge realms are needed (Olazabal et al. 2021). Integrating specialised technical knowledge and making this actionable is a key challenge in current urban governance aiming towards greener and smarter city concepts (Plitt et al. 2022). Engaging stakeholders across sectors and knowledge types could facilitate collaborative learning and help addressing symptoms and causes of vulnerability (Werners et al. 2021; Kabisch et al. 2022). This way, NbS management can navigate closer towards environmental justice considerations (Pineda-Pinto et al. 2021).

For technology to help arrive at fairer adaptation measures, it is critical that the tools have low access barriers and are complemented with off-line measures (Plitt et al. 2022). Informative and understandable visualisation capabilities are needed for tools to serve as discussion bases (see “Linking SETS dimensions to reinforce NbS” section for details). Tools can support visioning, scenario and target development incorporating diverse knowledge (Plitt et al. 2022). One helpful tool is generic internet forum software providing discussion threads and private messages allowing creation of connections between previously unconnected stakeholders (Afzalan and Muller 2014).

However, adding technology to decision-making processes also introduces novel challenges compared to more classical governance approaches (Gulsrud et al. 2018; Toxopeus et al. 2020; Li and Nassauer 2021). Therefore, IT cannot be regarded as a solution per se, but as an extension to current approaches with the goal of increasing connections in society (Møller et al. 2019). As digital tools and digital data exclude a certain part of society (Afzalan and Muller 2018), they are not meant to replace existing democratically backed forms of governance. Frameworks need to be in place to encounter tendencies of exclusion (Gulsrud et al. 2018). Further challenges arise from potentially decoupling people and place by introducing generalised systems that are not adapted to local circumstances (Pan et al. 2022). Such forms of delocalisation, and potentially also depoliticization, need to be countered and avoided when integrating technology into NbS (Gulsrud et al. 2018; Finewood et al. 2019).

Overall, newly formed decentral NbS need less technocracy than classic grey solutions (Dhakal and Chevalier 2016). Instead, they need specific and carefully mediated connections between stakeholders. To democratise NbS, virtual and physical places must be created for mutual discussions to profit from the diversity inside the urban system. Such meetings could be taking place in facilities provided by the city. Barriers to entry need to be very low-threshold, and digital tools—if used—should ideally be complemented with offline and non-digital approaches. Working in this direction, the concept of our developed interface will be tested and fine-tuned together with stakeholders and managers around NbS in future projects.

### Environmental monitoring data as a component of SET-interfaces

Besides the usability of the tools, what data they portray is essential (Ugolini et al. 2015). Performance or state data need to be contextualized and connected to ecosystem service provisioning and management options. Temporally continuous information is useful when dealing with immediate, lagged or long-term gradual effects of climate change (Elmqvist et al. 2018). While intense, short-term disturbances and their influences might get wide media coverage and attention from a diverse stakeholder group, other disturbances are more difficult to detect, analyse, and deal with. Hence, data need to allow for spatially and temporally explicit performance and impact evaluation. Therefore, sensor data of places known to people can elevate discussions regarding climate change or the role of trees in cities (Afzalan and Muller 2018) making monitoring technologies and the data they produce valuable (Grêt-Regamey et al. 2021).

So far, monitoring approaches for NbS are underdeveloped and underused, especially regarding their multifunctionality (Chrysoulakis et al. 2021; Kumar et al. 2021). The lack of monitoring can partially be attributed to a lack of concepts and knowledge in the scientific community, which often stops at the level of data generation, producing neither actionable nor accessible information resulting in products that are of limited use for application (see two recent reviews on this topic for the field of remote sensing: Wellmann et al., 2020; Zhu et al., 2019).

Remote sensing data are covering large areas and time frames and are available globally (Lausch et al. 2018). Therefore, remote sensing enables spatio-temporally consistent analysis that can help overcome boundaries caused by differing timings in ecological and social systems, disentangling gradual, sudden, or repetitive signals, and might limit spatial misrepresentations caused by social biases. It can further help to make ecosystem service models more spatially accurate or upscale developed methods (Pan et al. 2021). Setting remotely sensed ecological indicators into social context and vice versa, environmental data can help evaluate adaptation measures and expand knowledge exchange around NbS management in urban environments (Endreny 2018; Dumitru and Wendling 2021). Such developments can set the direction for the integration of other kinds of monitoring data into participatory planning support systems (Afzalan and Muller 2018; Pan et al. 2022).

Sensors always have to be checked and the information they produce validated, in a best-case scenario directly by citizens further spurring direct involvement (Kahila-Tani et al. 2016). In optical remote sensing the derived information stems from statistic- or mechanistic models as the object’s characteristics are not directly—but remotely—studied. While leaf traits like chlorophyll or nitrogen concentration are well detectable in many circumstances, there are certainly traits that show weaker correlation and are more difficult to sense remotely (Boegh et al. 2002; Wang et al. 2005). These can be chemical components where absorption bands are subtler or in regions of the electromagnetic spectrum not covered by multispectral satellite sensors. Traits like leaf dry-matter content are based on multiple spectral characteristics and can thus be sensed by hyperspectral sensing methods with much higher accuracy (Wang et al. 2011). Therefore, integrating such sensors in NbS management is desirable to support multispectral satellite sensors with infrequent additional information. We suggest to follow this route to improve the indicators portrayed in this study. This is especially important, as in multi-stakeholder systems the trustworthiness of data must be very high.

### Trait diversity as a component of SET-interfaces

Climatic extremes will likely increase in magnitude and frequency in the future, increasing the uncertainty for urban systems (Dodman et al. 2022). This requires a strategy of creating structures that are rooted in functional diversity, as it remains largely unknown which trees will flourish under different climatic conditions in 50 years’ time in cities (c.f. an example for central European cities by Schönfeld, 2019). Therefore, working with redundancy as a strategy of amplifying response diversity though different NbS types, different functional ecological components in them and diverse support systems can increase climate resistance and thus resilience.

In this direction, linking ecosystem services and people’s preferences to traits can be a component of social-ecological interfaces breaking down functional diversity in NbS. On the ecological side, it can be shown that NbS policies regarding functional traits promote greater resistance to climate change (Paquette et al. 2021). Further, traits have the potential to serve as a boundary object for sense and decision-making (Andersson et al. 2021). This can for instance be the detection of visible changes in leaf and canopy traits due to heat stress representing losses in fitness and ecosystem services (see Fig. 1 as an example). Hence, traits can be a way of (re)constructing healthy ecosystems in cities for people and nature through adaptive and integrative management (Andersson et al. 2021). Critical for such a form of management is to find traits, meaningful for ecological processes as well as for social considerations such as ecosystem services.

The traits we analysed in this study—vegetation density of the canopy and its photosynthetic activeness—are related to the primary functions and services that urban trees provide, i.e., cooling, shade provisioning, or noise reduction, which are highly valued ecosystem services (Palliwoda and Priess 2021). Some traits and functions in this direction could be monitored by a broad group of stakeholders. At the same time, we show that those traits are also globally analysable though remote sensing monitoring.

## Conclusions

Cities need additional solutions to respond—in different and complementary ways—to climate change. NbS is a many-faceted, systemic approach that seeks to do this by creating and maintaining green and blue structures essential for human wellbeing. Yet these structures themselves are threatened by climate change, as our case of ecosystem service provision by urban trees during a hot drought shows. To capture and address this duality, we searched for linked social-ecological-technological approaches to incorporate into the design of a prototype for NbS planning and management support, primed for urban trees and drought.

The focused review highlighted the potential in using tailored IT products to promote stronger connections between SETS dimensions. We elicited three guiding principles for designing SETS-interfaces, namely providing feedback, common ground, and diverse knowledge integration. We put forth three suggestions how IT products can live up to those principles: contextualisation of indicators to relate the functioning of NbS to the local problem context they seek to solve, good visualisation capabilities, and usage of trustworthy information based on up-to-date data. This way technology can be a valuable asset helping to connect stakeholders, distributing evidence, and spurring visioning and discussion. Towards this, local guidance is needed to steer the process and make sure that presented information is trustworthy.

To demonstrate the principles, we developed a prototype interface tailored to the Leipzig show case. The prototype combines monitoring information of vegetation diversity and performance with socio-demographic data relevant for public health to prioritise different climate change adaptation pathways locally. It demonstrates how to set indicators of different SET-dimensions into context, here we used vulnerability and density in population and show how these two variables can impact adaptation goals and the distribution of actions. Remote sensing helps to add spatially and temporally explicit information, allowing managers to track functioning and non-functioning of NbS and make management visible, without installing local hardware. Monitoring information makes faster assessment cycles in changing conditions possible. As NbS performance must be measured in relation to what type of solution the NbS is meant to provide, we suggest place-based, integrated evidence bases are needed allowing to tailor generalist policy goals to local settings. Taking such a route, data and tools can become meaningful in management and could help to reinforce the capacity of NbS to be actual solutions.

## Supplementary Information

Below is the link to the electronic supplementary material.

Electronic supplementary material 1 (PDF 1190 kb)

## References

[CR1] Afzalan N, Muller B (2014). The role of social media in green infrastructure planning: A case study of neighborhood participation in park siting. Journal of Urban Technology.

[CR2] Afzalan N, Muller B (2018). Online participatory technologies: Opportunities and challenges for enriching participatory planning. Journal of the American Planning Association.

[CR3] Almenar JB, Elliot T, Rugani B, Philippe B, Gutierrez TN, Sonnemann G, Geneletti D (2021). Nexus between nature-based solutions, ecosystem services and urban challenges. Land Use Policy.

[CR4] Andersson E, Barthel S, Borgström S, Colding J, Elmqvist T, Folke C, Gren Å (2014). Reconnecting cities to the biosphere: Stewardship of green infrastructure and urban ecosystem services. Ambio.

[CR5] Andersson E, Borgström S, McPhearson T, Kabisch N, Korn H, Stadler J, Bonn A (2017). Double insurance in dealing with extremes: Ecological and social factors for making nature-based solutions last. Nature-based solutions to climate change adaptation in urban areas.

[CR6] Andersson E, Langemeyer J, Borgström S, McPhearson T, Haase D, Kronenberg J, Barton DN, Davis M (2019). Enabling green and blue infrastructure to improve contributions to human well-being and equity in urban systems. BioScience.

[CR7] Andersson E, Haase D, Scheuer S, Wellmann T (2020). Neighbourhood character affects the spatial extent and magnitude of the functional footprint of urban green infrastructure. Landscape Ecology.

[CR8] Andersson E, Haase D, Andersson P, Cortinovis C, Goodness J, Kendal D, Lausch A, McPhearson T (2021). What are the traits of a social-ecological system? Towards a framework in support of urban sustainability. Npj Urban Sustainability.

[CR9] Bellamy C, van der Jagt A, Barbour S, Smith M, Moseley D (2017). A spatial framework for targeting urban planning for pollinators and people with local stakeholders: A route to healthy, blossoming communities?. Environmental Research.

[CR10] de Bello F, Lavorel S, Hallett L, Valencia E, Garnier E, Roscher C, Conti L, Galland T (2021). Functional trait effects on ecosystem stability: Assembling the jigsaw puzzle. Trends in Ecology & Evolution.

[CR11] Boegh E, Soegaard H, Broge N, Hasager CB, Jensen NO, Schelde K, Thomsen A (2002). Airborne multispectral data for quantifying leaf area index, nitrogen concentration, and photosynthetic efficiency in agriculture. Remote Sensing of Environment.

[CR12] Brink E, Aalders T, Ádám D, Feller R, Henselek Y, Hoffmann A, Ibe K, Matthey-Doret A (2016). Cascades of green: A review of ecosystem-based adaptation in urban areas. Global Environmental Change.

[CR13] Buurman J, Mens MJP, Dahm RJ (2017). Strategies for urban drought risk management: A comparison of 10 large cities. International Journal of Water Resources Development.

[CR14] Chrysoulakis N, Somarakis G, Stagakis S, Mitraka Z, Wong M (2021). Monitoring and evaluating nature-based solutions implementation in urban areas by means of earth observation. Remote Sensing.

[CR15] Cohen-Shacham E, Walters G, Janzen C, Maginnis S (2016). Nature-based solutions to address global societal challenges. *IUCN: Gland*. Switzerland.

[CR16] Croeser, T., G. E. Garrard, F. M. Thomas, T. D. Tran, I. Mell, S. Clement, R. Sánchez, and S. Bekessy. 2021. Diagnosing delivery capabilities on a large international nature-based solutions project. *npj Urban Sustainability* 1: 1–9.

[CR17] Deutscher Wetterdienst. 2022. Climate data Germany (Klimadaten Deutschland). www.dwd.de/DE/leistungen/klimadatendeutschland/klimadatendeutschland.html. Accessed 10 Aug 2022.

[CR18] Dhakal KP, Chevalier LR (2016). Urban stormwater governance: The need for a paradigm shift. Environmental Management.

[CR19] Díaz S, Cabido M (2001). Vive la différence: Plant functional diversity matters to ecosystem processes. Trends in Ecology and Evolution.

[CR20] Dodman, D. M., B. Hayward, M. Pelling, V. Castan Broto, W. Chow, E. Chu, R. Dawson, L. Khirfan, et al. 2022. Cities, settlements and key infrastructure. In *Climate change 2022: Impacts, adaptation and vulnerability. Contribution of Working Group II to the sixth assessment report of the intergovernmental panel on climate change*, ed. H. Pörtner, D. Roberts, M. Tignor, E. Poloczanska, K. Mintenbeck, A. Alegria, M. Craig, S. Langsdorf, et al., 907–1040. Cambridge, UK and New York, NY, USA: Cambridge University Press.

[CR21] Dumitru A, Wendling L (2021). Evaluating the impact of nature-based solutions: A handbook for practitioners.

[CR22] Dumitru A, Frantzeskaki N, Collier M (2020). Identifying principles for the design of robust impact evaluation frameworks for nature-based solutions in cities. Environmental Science and Policy.

[CR23] Egerer, M., D. Haase, T. McPhearson, N. Frantzeskaki, E. Andersson, H. Nagendra, and A. Ossola. 2021. Urban change as an untapped opportunity for climate adaptation. *npj Urban Sustainability* 1: 1–9.

[CR24] Elmqvist T, Siri J, Andersson E, Anderson P, Bai X, Das PK, Gatere T, Gonzalez A (2018). Urban tinkering. Sustainability Science.

[CR25] Endreny TA (2018). Strategically growing the urban forest will improve our world. Nature Communications.

[CR26] Fastenrath S, Bush J, Coenen L (2020). Scaling-up nature-based solutions. Lessons from the Living Melbourne strategy. Geoforum.

[CR27] Finewood MH, Matsler AM, Zivkovich J (2019). Green infrastructure and the hidden politics of urban stormwater governance in a postindustrial city. Annals of the American Association of Geographers.

[CR28] Fu X, Goddard H, Wang X, Hopton ME (2019). Development of a scenario-based stormwater management planning support system for reducing combined sewer overflows (CSOs). Journal of Environmental Management.

[CR29] Grêt-Regamey, A., M. Switalski, N. Fagerholm, S. Korpilo, S. Juhola, M. Kyttä, N. Käyhkö, T. McPhearson, et al. 2021. Harnessing sensing systems towards urban sustainability transformation. *npj Urban Sustainability* 1: 1–9.

[CR30] Gulsrud NM, Raymond CM, Rutt RL, Olafsson AS, Plieninger T, Sandberg M, Beery TH, Jönsson KI (2018). ‘Rage against the machine’? The opportunities and risks concerning the automation of urban green infrastructure. Landscape and Urban Planning.

[CR31] Haase D, Hellwig R (2022). Trees, forests and people effects of heat and drought stress on the health status of six urban street tree species in Leipzig, Germany. Trees, Forests and People.

[CR32] Haralick R, Shanmugam K, Dinstein I (1973). Textural features for image classification. IEEE Transactions on Systems, Man and Cybernetics.

[CR33] Hasala D, Supak S, Rivers L (2020). Green infrastructure site selection in the Walnut Creek wetland community: A case study from southeast Raleigh, North Carolina. Landscape and Urban Planning.

[CR34] Homolová L, Malenovský Z, Clevers J, García-Santos G, Schaepman M (2013). Review of optical-based remote sensing for plant trait mapping. Ecological Complexity.

[CR35] Kabisch N, Frantzeskaki N, Pauleit S, Naumann S, Davis M, Artmann M, Haase D, Knapp S (2016). Nature-based solutions to climate change mitigation and adaptation in urban areas: Perspectives on indicators, knowledge gaps, barriers, and opportunities for action. Ecology and Society.

[CR36] Kabisch N, Frantzeskaki N, Hansen R (2022). Principles for urban nature-based solutions. Ambio.

[CR37] Kahila-Tani M, Broberg A, Kyttä M, Tyger T (2016). Let the citizens map—public participation GIS as a planning support system in the Helsinki master plan process. Planning Practice & Research.

[CR38] Kattge J, Bönisch G, Diaz S, Lavorel S, Prentice IC, Leadley P, Tautenhahn S, Werner GDA (2020). TRY plant trait database–enhanced coverage and open access. Global Change Biology.

[CR39] Konijnendijk CC, Ricard RM, Kenney A, Randrup TB (2006). Defining urban forestry–A comparative perspective of North America and Europe. Urban Forestry & Urban Greening.

[CR40] Kumar P, Debele SE, Sahani J, Rawat N, Marti-cardona B, Maria S, Basu B, Sarkar A (2021). An overview of monitoring methods for assessing the performance of nature-based solutions against natural hazards. Earth-Science Reviews.

[CR41] Lausch A, Bastian O, Herzog F, Leitão P, Rocchini D, Tischendorf L, Olaf B, Stefan K (2018). Understanding and assessing vegetation health by in-situ species and remote sensing approaches. Methods in Ecology and Evolution.

[CR42] Leonard L, Miles B, Heidari B, Lin L, Castronova AM, Minsker B, Lee J, Scaife C (2019). Development of a participatory Green Infrastructure design, visualization and evaluation system in a cloud supported jupyter notebook computing environment. Environmental Modelling and Software.

[CR43] Li J, Nassauer JI (2021). Technology in support of nature-based solutions requires understanding everyday experiences. Ecology and Society.

[CR44] Lin BB, Ossola A, Alberti M, Andersson E, Bai X, Dobbs C, Elmqvist T, Evans KL (2021). Integrating solutions to adapt cities for climate change. The Lancet Planetary Health.

[CR45] Lloret F, Lobo A, Estevan H, Maisongrande P, Vayreda J, Terradas J (2007). Woody plant richness and NDVI response to drought events in Catalonian (Northeastern Spain) forests. Ecology.

[CR46] Marselle MR, Bowler DE, Watzema J, Eichenberg D, Kirsten T, Bonn A (2020). Urban street tree biodiversity and antidepressant prescriptions. Scientific Reports.

[CR47] McPhearson T, Cook EM, Berbés-Blázquez M, Cheng C, Grimm NB, Andersson E, Barbosa O, Chandler DG (2022). A social-ecological-technological systems framework for urban ecosystem services. One Earth.

[CR48] Meerow S, Newell JP, Stults M (2016). Defining urban resilience: A review. Landscape and Urban Planning.

[CR49] Møller MS, Olafsson AS (2018). The use of e-tools to engage citizens in urban green infrastructure governance: Where do we stand and where are we going?. Sustainability.

[CR50] Møller MS, Olafsson AS, Vierikko K, Sehested K, Elands B, Buijs A, van den Bosch CK (2019). Participation through place-based e-tools: A valuable resource for urban green infrastructure governance?. Urban Forestry and Urban Greening.

[CR51] Nash C, Clough J, Gedge D, Lindsay R, Newport D, Ciupala MA, Connop S (2016). Initial insights on the biodiversity potential of biosolar roofs: A London Olympic Park green roof case study. Israel Journal of Ecology and Evolution.

[CR52] Nayak SG, Shrestha S, Kinney PL, Ross Z, Sheridan SC, Pantea CI, Hsu WH, Muscatiello N (2018). Development of a heat vulnerability index for New York State. Public Health.

[CR53] Nesshöver C, Assmuth T, Irvine KN, Rusch GM, Waylen KA, Delbaere B, Haase D, Jones-Walters L (2017). The science, policy and practice of nature-based solutions: An interdisciplinary perspective. Science of the Total Environment.

[CR54] Newton P, Frantzeskaki N (2021). Creating a national urban research and development platform for advancing urban experimentation. Sustainability (switzerland).

[CR55] Nock CA, Paquette A, Follett M, Nowak DJ, Messier C (2013). Effects of urbanization on tree species functional diversity in Eastern North America. Ecosystems.

[CR56] O’Donnell EC, Lamond JE, Thorne CR (2018). Learning and Action Alliance framework to facilitate stakeholder collaboration and social learning in urban flood risk management. Environmental Science and Policy.

[CR57] Olazabal M, Chu E, Broto VC, Patterson J (2021). Subaltern forms of knowledge are required to boost local adaptation. One Earth.

[CR58] Othengrafen F, Levin-Keitel M (2019). Planners between the chairs: How planners (do not) adapt to transformative practices. Urban Planning.

[CR59] Palliwoda J, Priess JA (2021). What do people value in urban green? Linking characteristics of urban green spaces to users’ perceptions of nature benefits, disturbances, and disservices. Ecology and Society.

[CR60] Palliwoda J, Banzhaf E, Priess JA (2020). How do the green components of urban green infrastructure influence the use of ecosystem services? Examples from Leipzig, Germany. Landscape Ecology.

[CR61] Pan H, Page J, Cong C, Barthel S, Kalantari Z (2021). How ecosystems services drive urban growth: Integrating nature-based solutions. Antropocene.

[CR62] Pan H, Kwak Y, Deal B (2022). Participatory development of planning support systems to improve empowerment and localization. Journal of Urban Technology.

[CR63] Paquette A, Sousa-Silva R, Maure F, Cameron E, Belluau M, Messier C (2021). Praise for diversity: A functional approach to reduce risks in urban forests. Urban Forestry & Urban Greening.

[CR64] Partelow S, Winkler KJ (2016). Interlinking ecosystem services and Ostrom’s framework through orientation in sustainability research. Ecology and Society.

[CR65] Pineda-Pinto M, Frantzeskaki N, Nygaard CA (2021). The potential of nature-based solutions to deliver ecologically just cities: Lessons for research and urban planning from a systematic literature review. Ambio.

[CR66] Planet Labs. 2022. *Planet Imagery Product Specifications*. www.assets.planet.com/docs/Planet_Combined_Imagery_Product_Specs_letter_screen.pdf Accessed 05 June 2022

[CR67] Plitt S, Johnson M, Andersson E (2022). Assessing the potential of E-tools for knowledge sharing and stewardship of urban green infrastructure. Arboriculture & Urban Forestry.

[CR68] Rink, D., and C. Schmidt. 2021. Afforestation of urban brownfields as a nature-based solution. Experiences from a project in Leipzig (Germany). *Land* 10: 893.

[CR69] Scheuer S, Haase D, Haase A, Wolff M, Wellmann T (2021). A glimpse into the future of exposure and vulnerabilities in cities? Modelling of residential location choice of urban population with random forest. Natural Hazards and Earth System Sciences.

[CR70] Schneider F, Morsdorf F, Schmid B, Petchey O, Hueni A, Schimel D, Schaepman M (2017). Mapping functional diversity from remotely sensed morphological and physiological forest traits. Nature Communications.

[CR71] Schönfeld, P. 2019. “Climate trees”—which species can be planted in the future? (In German). *LWG aktuell*: 1–9.

[CR72] Shahtahmassebi AR, Li C, Fan Y, Wu Y, Gan M, Wang K, Malik A, Blackburn GA (2021). Remote sensing of urban green spaces: A review. Urban Forestry & Urban Greening.

[CR73] Sjöman H, Hirons AD, Bassuk NL (2018). Improving confidence in tree species selection for challenging urban sites: A role for leaf turgor loss. Urban Ecosystems.

[CR74] Somarakis, G., S. Stagakis, and N. Chrysoulakis, ed. 2019. *ThinkNature Nature-Based Solutions Handbook*. ThinkNature project funded by the EU Horizon 2020 research and innovation programme under grant agreement No. 730338. 10.26225/jerv-w202.

[CR75] Stadt Leipzig. Open Data Leipzig, Available at: www.opendata.leipzig.de. 10-Sept-2021.

[CR76] Taylor JR, Hanumappa M, Miller L, Shane B, Richardson ML (2021). Facilitating multifunctional green infrastructure planning in washington, dc through a tableau interface. Sustainability.

[CR77] Tengö M, Hill R, Malmer P, Raymond CM, Spierenburg M, Danielsen F, Elmqvist T, Folke C (2017). Weaving knowledge systems in IPBES, CBD and beyond—lessons learned for sustainability. Current Opinion in Environmental Sustainability.

[CR78] Toxopeus H, Kotsila P, Conde M, Katona A, van der Jagt APN, Polzin F (2020). How ‘just’ is hybrid governance of urban nature-based solutions?. Cities.

[CR79] Tzoulas K, Galan J, Venn S, Dennis M, Pedroli B, Mishra H, Haase D, Pauleit S (2021). A conceptual model of the social–ecological system of nature-based solutions in urban environments. Ambio.

[CR80] Ugolini F, Massetti L, Sanesi G, Pearlmutter D (2015). Knowledge transfer between stakeholders in the field of urban forestry and green infrastructure: Results of a European survey. Land Use Policy.

[CR81] Venter ZS, Barton DN, Martinez-Izquierdo L, Langemeyer J, Baró F, McPhearson T (2021). Interactive spatial planning of urban green infrastructure—Retrofitting green roofs where ecosystem services are most needed in Oslo. Ecosystem Services.

[CR82] Violle C, Navas M, Vile D, Kazakou E, Fortunel C, Hummel I, Garnier E (2007). Let the concept of trait be functional!. Oikos.

[CR83] Wang L, Qu JJ, Hao X, Hunt ER (2011). Estimating dry matter content from spectral reflectance for green leaves of different species. International Journal of Remote Sensing.

[CR84] Wang Q, Adiku S, Tenhunen J, Granier A (2005). On the relationship of NDVI with leaf area index in a deciduous forest site. Remote Sensing of Environment.

[CR85] Watkins H, Hirons A, Sjöman H, Cameron R, Hitchmough JD (2021). Can trait-based schemes be used to select species in urban forestry?. Frontiers in Sustainable Cities.

[CR86] Weise H, Auge H, Baessler C, Bärlund I, Bennett EM, Berger U, Bohn F, Bonn A (2020). Resilience trinity: Safeguarding ecosystem functioning and services across three different time horizons and decision contexts. Oikos.

[CR87] Wellmann T, Haase D, Knapp S, Salbach C, Selsam P, Lausch A (2018). Urban land use intensity assessment: The potential of spatio-temporal spectral traits with remote sensing. Ecological Indicators.

[CR88] Wellmann T, Lausch A, Andersson E, Knapp S, Cortinovis C, Jache J, Scheuer S, Kremer P (2020). Remote sensing in urban planning: Contributions towards ecologically sound policies?. Landscape and Urban Planning.

[CR89] Werners SE, Wise RM, Butler JRA, Totin E, Vincent K (2021). Adaptation pathways: A review of approaches and a learning framework. Environmental Science and Policy.

[CR90] Wickenberg B, McCormick K, Olsson JA (2021). Advancing the implementation of nature-based solutions in cities: A review of frameworks. Environmental Science and Policy.

[CR91] Xie C, Wang J, Haase D, Wellmann T, Lausch A (2023). Measuring spatio-temporal heterogeneity and interior characteristics of green spaces in urban neighborhoods: A new approach using gray level co-occurrence matrix. Science of the Total Environment.

[CR92] Yang J, Yin P, Sun J, Wang B, Zhou M, Li M, Tong S, Meng B (2019). Heatwave and mortality in 31 major Chinese cities: Definition, vulnerability and implications. Science of the Total Environment.

[CR93] Zhang Y, Migliavacca M, Penuelas J, Ju W (2021). Advances in hyperspectral remote sensing of vegetation traits and functions. Remote Sensing of Environment.

[CR94] Zheng Z, Zeng Y, Schneider FD, Zhao Y, Zhao D, Schmid B, Schaepman ME, Morsdorf F (2020). Mapping functional diversity using individual tree-based morphological and physiological traits in a subtropical forest. Remote Sensing of Environment.

[CR95] Zhu Z, Zhou Y, Seto KC, Stokes EC, Deng C, Pickett STA, Taubenböck H (2019). Understanding an urbanizing planet: Strategic directions for remote sensing. Remote Sensing of Environment.

[CR96] Zingraff-Hamed A, Hüesker F, Albert C, Brillinger M, Huang J, Lupp G, Scheuer S, Schlätel M (2021). Governance models for nature-based solutions: Seventeen cases from Germany. Ambio.

