# Supplementary material for: Reinforcing nature-based solutions through tools providing social-ecological-technological integration
Source: Ambio. 2022 Oct 26;52(3):489–507. doi: 10.1007/s13280-022-01801-4 (PMC9849649; doi:10.1007/s13280-022-01801-4)
Supplement: Supplementary file 1 — Electronic supplementary material 1 (PDF 1190 kb) [file 13280_2022_1801_MOESM1_ESM.pdf]

Ambio

Supplementary Information

This supplementary information has not been peer reviewed.

Title: Reinforcing nature-based solutions through tools providing social-ecological-technological integration

Supplementary information

S1 Review type and goals

We performed a focused review, which is a type of review for asking questions in very specific contexts (Gordon et al., 2019). Those reviews can have narrow scopes following a question in a specific context for targeted learning outcomes. In our case, both the number in articles as well the fact that we only searched for positive examples limits the review in its universality (Gordon, et al. 2019). Still, the focused- approach can be useful “for initial work to obtain understanding of the issues relevant to the question” (RWE-Navigator, n.d.).

Based on these methods we ask for our review part: “Are there cases in the literature where information technology helped enable a broader stakeholder contribution around NbS and what can we learn for the development of our tool for reinforcing Leipzig’s NbS?”. It is not the intent to give a complete representation of the field but much rather a step in our learning towards developing a meaningful interface. We wanted to learn how information technology and environmental data were integrated across the studies to enable knowledge flows and communication.

S2 Database query

Generally, our review follows the PRISMA guidelines (Moher et al., 2009). For this, we used the SCOPUS database, being the largest database of peer reviewed journal articles (Mongeon & Paul-Hus, 2016). Herein we searched for journal articles in English containing a combination of the following search terms in their respective title, abstract or keywords (a * was used to incorporate different forms of the respective search term):

```
TITLE-ABS-KEY   (
                (urban OR city)
          AND   (citizen* OR society OR stakeholder OR community ")
          AND   (nbs OR "green infrastructure")
          AND   (technological OR technology OR App OR platform OR "geographic information system"
                OR "web application" OR "online application")
          AND   (engag* OR coordinati* OR participat* OR enabl* OR empower* OR exchang* OR
          shar*)
        )
          AND   (LIMIT-TO (SRCTYPE , "j" ))
          AND   (LIMIT-TO (DOCTYPE , "ar" ))
          AND   (LIMIT-TO (LANGUAGE , "English" ))
```

Thematically we limited our search to studies coping with nature-based solutions (NbS) and green infrastructure in an urban social–ecological–technological systems (SETS) context reporting on desirable outcomes like engagement, participation, empowerment etc. The query was performed on the first of November 2021.

S3 Query results

The query resulted in 29 articles. Of those, two were unavailable to the authors and seven did not match the scope of this research after full text screening (see table 1 for bibliographical details and reasons for exclusion). This resulted in 20 articles eligible for analysis.

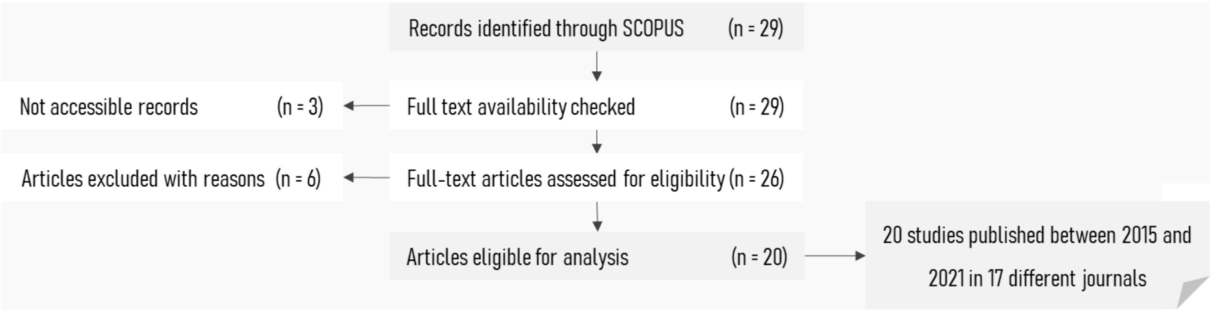

Figure S1. PRISMA flowchart

Table S1. Bibliographical details of the literature found through the SCOPUS literature search with information on availability and reasons for exclusion if applicable sorted by year of publication from the review.

Authors	Title	Year	Journal	Available?	Reason for exclusion
Taylor, J.R., Hanumappa, M., Miller, L., Shane, B., Richardson, M.L.	Facilitating multifunctional green infrastructure planning in washington, dc through a tableau interface	2021	Sustainability (Switzerland)	Yes	
Venter, Z.S., Barton, D.N., Martinez-Izquierdo, L., Langemeyer, J., Baró, F., McPhearson, T.	Interactive spatial planning of urban green infrastructure – Retrofitting green roofs where ecosystem services are most needed in Oslo	2021	Ecosystem Services	Yes	
Newton, P., Frantzeskaki, N.	Creating a national urban research and development platform for advancing urban experimentation	2021	Sustainability (Switzerland)	Yes	
Restemeyer, B., Boogaard, F.C.	Potentials and pitfalls of mapping nature-based solutions with the online citizen science platform climatescan	2021	Land	Yes	
Fastenrath, S., Bush, J., Coenen, L.	Scaling-up nature-based solutions. Lessons from the Living Melbourne strategy	2020	Geoforum	Yes	
Hasala, D., Supak, S., Rivers, L.	Green infrastructure site selection in the Walnut Creek wetland community: A case study from southeast Raleigh, North Carolina	2020	Landscape and Urban Planning	Yes	
Zuniga-Teran, A.A., Staddon, C., de Vito, L., Gerlak, A.K., Ward, S., Schoeman, Y., Hart, A., Booth, G.	Challenges of mainstreaming green infrastructure in built environment professions	2020	Journal of Environmental Planning and Management	Yes	
Mussinelli, E., Tartaglia, A., Castaldo, G., Fanzini, D.	The environmental technological giovannicastaido project for the implementation of the 2030 agenda [Il progetto tecnologico ambientale per l'attuazionedell'agenda 2030]	2020	Sustainable Mediterranean Construction	No	Not available
Finewood, M.H., Matsler, A.M., Zivkovich, J.	Green Infrastructure and the Hidden Politics of Urban Stormwater Governance in a Postindustrial City	2019	Annals of the American Association of Geographers	Yes	
Fu, X., Goddard, H., Wang, X., Hopton, M.E.	Development of a scenario-based stormwater management planning support system for reducing combined sewer overflows (CSOs)	2019	Journal of Environmental Management	Yes	
Møller, M.S., Olafsson, A.S., Vierikko, K., Sehested, K., Elands, B., Buijs, A., van den Bosch, C.K.	Participation through place-based e-tools: A valuable resource for urban green infrastructure governance?	2019	Urban Forestry and Urban Greening	Yes	
Bąk, J., Królikowska, J.	Current status and possibilities of implementing green walls for adaptation to climate change of urban areas on the example of Krakow [Stan obecny i możliwości implementacji zielonych ścian w celu adaptacji do zmian klimatu terenów zurbanizowanych na przykładzie miasta Krakowa]	2019	Rocznik Ochrona Srodowiska	No	Not available
Atkinson, A.J., Apul, O.G., Schneider, O., Garcia-Segura, S., Westerhoff, P.	Nanobubble Technologies Offer Opportunities to Improve Water Treatment	2019	Accounts of Chemical Research	Yes	NBS = nanobubbles. Therefore, out of scope.
Leonard, L., Miles, B., Heidari, B., Lin, L., Castronova, A.M., Minsker, B., Lee, J., Scaife, C., Band, L.E.	Development of a participatory Green Infrastructure design, visualization and evaluation system in a cloud supported jupyter notebook computing environment	2019	Environmental Modelling and Software	Yes	

Gulsrud, N.M., Raymond, C.M., Rutt, R.L., Olafsson, A.S., Plieninger, T., Sandberg, M., Beery, T.H., Jönsson, K.I.	‘Rage against the machine’? The opportunities and risks concerning the automation of urban green infrastructure	2018	Landscape and Urban Planning	Yes	
Møller, M.S., Olafsson, A.S.	The use of e-tools to engage citizens in urban green infrastructure governance: Where do we stand and where are we going?	2018	Sustainability (Switzerland)	Yes	
Gulsrud, N.M., Hertzog, K., Shears, I.	Innovative urban forestry governance in Melbourne?: Investigating “green placemaking” as a nature-based solution	2018	Environmental Research	No	Not available
O'Donnell, E.C., Lamond, J.E., Thorne, C.R.	Learning and Action Alliance framework to facilitate stakeholder collaboration and social learning in urban flood risk management	2018	Environmental Science and Policy	Yes	
Raymond, C.M., Frantzeskaki, N., Kabisch, N., Berry, P., Breil, M., Nita, M.R., Geneletti, D., Calfapietra, C.	A framework for assessing and implementing the co-benefits of nature-based solutions in urban areas	2017	Environmental Science and Policy	Yes	Review without single case studies being presented
Bellamy, C.C., van der Jagt, A.P.N., Barbour, S., Smith, M., Moseley, D.	A spatial framework for targeting urban planning for pollinators and people with local stakeholders: A route to healthy, blossoming communities?	2017	Environmental Research	Yes	
Nash, C., Clough, J., Gedge, D., Lindsay, R., Newport, D., Ciupala, M.A., Connop, S.	Initial insights on the biodiversity potential of biosolar roofs: a London Olympic Park green roof case study	2016	Israel Journal of Ecology and Evolution	Yes	
Dhakal, K.P., Chevalier, L.R.	Urban Stormwater Governance: The Need for a Paradigm Shift	2016	Environmental Management	Yes	
Ávila, C., García, J., Garfí, M.	Influence of hydraulic loading rate, simulated storm events and seasonality on the treatment performance of an experimental three-stage hybrid CW system	2016	Ecological Engineering	Yes	No coupling between SETS domains
Guerrero, P., Møller, M.S., Olafsson, A.S., Snizek, B.	Revealing cultural ecosystem services through instagram images: The potential of social media volunteered geographic information for urban green infrastructure planning and governance	2016	Urban Planning	Yes	
Ugolini, F., Massetti, L., Sanesi, G., Pearlmutter, D.	Knowledge transfer between stakeholders in the field of urban forestry and green infrastructure: Results of a European survey	2015	Land Use Policy	Yes	
Afzalan, N., Muller, B.	The Role of Social Media in Green Infrastructure Planning: A Case Study of Neighborhood Participation in Park Siting	2014	Journal of Urban Technology	Yes	
Montalto, F.A., Bartrand, T.A., Waldman, A.M., Travaline, K.A., Loomis, C.H., McAfee, C., Geldi, J.M., Riggall, G.J., Boles, L.M.	Decentralised green infrastructure: The importance of stakeholder behaviour in determining spatial and temporal outcomes	2013	Structure and Infrastructure Engineering	Yes	No coupling between SETS domains
[No author name available]	8th International Conference on Urban Regeneration and Sustainability, SC 2013	2013	WIT Transactions on Ecology and the Environment	Yes	Series of abstracts, not a journal article
[No author name available]	8th International Conference on Urban Regeneration and Sustainability, SC 2013	2013	WIT Transactions on Ecology and the Environment	Yes	Series of abstracts, not a journal article

S4 Analysis protocol

Relevant articles were analysed using a structured review protocol. This framework consisted of three sections. The first of which retrieved general information regarding the article. The second part collects information regarding the concepts describing social, ecological, and technological considerations. The third part collects evidence on the type and success of a potential linking entity proposed in the article and and what additional pathways could become feasible by introducing the researched matter.

Section 1: General information

- Bibliographical details
- Availability statement
- Reason for exclusion

Section 2: SETS

- Which dimensions of the social–ecological–technological system where regarded?
- How was the social dimension characterised?
- How was the ecological dimension characterised?
- How was the technological dimension characterised?
- What solutions are presented that connect two or more of the regarded dimensions of the system?

Section 3: Pathways

- What was the goal of the study/the measure/the tool?
- Was this successful? If so, in which way? If not, why not?
- What were key features of the entity linking processes in the SETS that made them (un)successful?
- What are specific pathways that became reachable by the linking entity?

Literature in the supplementary information

Gordon, M., Grafton-Clarke, C., Hill, E., Gurbutt, D., Patricio, M., & Daniel, M. (2019). Twelve tips for undertaking a focused systematic review in medical education. *Medical Teacher*, *41*(11), 1232–1238.

Moher, D., Liberati, A., Tetzlaff, J., Altman, D. G., Altman, D., Antes, G., Atkins, D., Barbour, V., ..., & Tugwell, P. (2009). Preferred reporting items for systematic reviews and meta-analyses: The PRISMA statement. *PLoS Medicine*, *6*(7). https://doi.org/10.1371/journal.pmed.1000097

Mongeon, P., & Paul-Hus, A. (2016). The journal coverage of Web of Science and Scopus: a comparative analysis. *Scientometrics*, *106*(1), 213–228. https://doi.org/10.1007/s11192-015-1765-5

RWE-Navigator. (n.d.). *Conducting a literature review*. https://rwe-navigator.eu/using-the-navigator-decision-support-tool/clarify-the-issues/drivers-of-effectiveness/methods-to-explore-doe/literature-to-explore-or-identify-drivers-of-effectiveness/
